# Interfacial Behavior of Oligo(Ethylene Glycol) Dendrons Spread Alone and in Combination with a Phospholipid as Langmuir Monolayers at the Air/Water Interface

**DOI:** 10.3390/molecules24224114

**Published:** 2019-11-14

**Authors:** Da Shi, Dinh-Vu Nguyen, Mounir Maaloum, Jean-Louis Gallani, Delphine Felder-Flesch, Marie Pierre Krafft

**Affiliations:** 1 Institut Charles Sadron (CNRS), University of Strasbourg, 23 rue du Loess, 67034 Strasbourg CEDEX 2, France; da.shi@ics-cnrs.unistra.fr (D.S.); maaloum@unistra.fr (M.M.); 2 Institut de Physique et Chimie des Matériaux de Strasbourg (IPCMS, CNRS), University of Strasbourg, 23 rue du Loess. 67034 Strasbourg CEDEX 2, France; dinh-vu.nguyen@alumni.chimie-paristech.fr (D.-V.N.); gallani@ipcms.unistra.fr (J.-L.G.); delphine.felder@ipcms.unistra.fr (D.F.-F.)

**Keywords:** fluorocarbon, interfacial film, Brewster angle microscopy, atomic force microscopy, molecular recognition

## Abstract

Dendrons consisting of two phosphonate functions and three oligo(ethylene glycol) (OEG) chains grafted on a central phenoxyethylcarbamoylphenoxy group were synthesized and investigated as Langmuir monolayers at the surface of water. The OEG chain in the *para* position was grafted with a *t*-Bu end-group, a hydrocarbon chain, or a partially fluorinated chain. These dendrons are models of structurally related OEG dendrons that were found to significantly improve the stability of aqueous dispersions of iron oxide nanoparticles when grafted on their surface. Compression isotherms showed that all OEG dendrons formed liquid-expanded Langmuir monolayers at large molecular areas. Further compression led to a transition ascribed to the solubilization of the OEG chains in the aqueous phase. Brewster angle microscopy (BAM) provided evidence that the dendrons fitted with hydrocarbon chains formed liquid-expanded monolayers throughout compression, whilst those fitted with fluorinated end-groups formed crystalline-like domains, even at large molecular areas. Dimyristoylphosphatidylcholine and dendron molecules were partially miscible in monolayers. The deviations to ideality were larger for the dendrons fitted with a fluorocarbon end-group chain than for those fitted with a hydrocarbon chain. Brewster angle microscopy and atomic force microscopy supported the view that the dendrons were ejected from the phospholipid monolayer during the OEG conformational transition and formed crystalline domains on the surface of the monolayer.

## 1. Introduction

Dendrimers are monodisperse macromolecules with a regular, highly branched, and well defined three-dimensional architecture, which have garnered interest in nanotechnology, materials science, and medicine [1,2,3]. In particular, they have been used in the synthesis and stabilization of metal nanoparticles used as drug delivery carriers and chemical and biomedical sensors [4]. Dendrons, which are fractional dendrimers, can assemble in solution to form a variety of nano- and microstructures [5,6], and have been found to form stable organized molecular films at the air/water interface [7]. Oligo(ethylene glycol) (OEG) dendrons have been used as efficient coating agents of iron oxide nanoparticles (IONPs) to improve the dispersibility and stability of these nanoparticles in aqueous solution [8,9]. Poly(ethylene glycol) (PEG) dendron-based phospholipids have been utilized for the preparation of stealth liposomes in biomedical applications [10]. Understanding the behavior of PEG and OEG dendrons is key to the development of interfacial applications [11,12]. The physicochemical properties of dendrons and, in particular, their interfacial behavior, strongly depend on their nature, generation number, and ratio of hydrophilic and hydrophobic parts [13,14]. Langmuir films are an effective tool for studying monolayers of amphiphilic dendrimers or dendrons at the air/water interface, which provide information such as dendron size, shape, and compressibility [11,15]. The Langmuir–Blodgett (LB) technique is commonly used for the fabrication of ordered monolayers by transfer onto solid substrate surfaces. Information about the microstructure of monolayers of dendrimers prepared by the LB technique has been obtained using X-ray, atomic force microscopy (AFM), Brewster angle microscopy (BAM), and neutron reflectivity [14,16,17,18].

Although numerous studies have reported on the behavior of dendrimers or dendrons at the air/water interface, few investigations have focused on the morphology and orientation of dendrons bearing OEG chains. One study of dendrons fitted with an OEG chain and poly(benzyl ether) (third to fifth generation) showed that stability of the monolayers increased with OEG chain length [7]. The stability of monolayers of OEGylated carbazole dendrons was found to depend mostly on the generation of the hydrophobic part [19]. The interfacial behavior of polyol–polyether dendritic amphiphiles fitted with two hydrocarbon chains has been examined [20], as well as the interactions of these dendrons with dipalmitoylphosphatidylcholine (DPPC) [21]. A compression isotherm and BAM study indicated that dendrons with the highest generation of polyglycerol form denser monolayers, and that they form mutually soluble binary mixtures with the phospholipid.

Here, we investigated the behavior of OEG-based dendrons at the air/water interface, and the behavior of combinations of these dendrons with dimyristoylphosphatidylcholine (DMPC). Our OEG dendrons consisted of a central phenoxyethylcarbamoyl group coupled to a phenyl group onto which three OEG chains were grafted (Frechet-type dendron), and bearing two phosphonate esters on the phenoxy group. These OEG dendrons are structurally close to the dendrons that have been found to improve the dispersibility and stability of iron oxide nanoparticles (IONPs) in aqueous solutions, a critical property for many studies and applications [8,22]. Recent work has established that IONPs grafted with OEG dendrons fitted with fluorinated end-groups form spontaneously adsorbed (Gibbs) films with low interfacial tension, especially when the atmosphere is saturated with a fluorocarbon [23]. As a consequence of this enhanced adsorption, small and stable fluorocarbon-stabilized microbubbles with a shell of phospholipid incorporating IONPs grafted with C_2_F_5_-terminated OEG dendrons were obtained [23]. The mixed DMPC/OEG dendron monolayers are indeed model interfaces that can provide insights into the interactions between the components, namely phospholipid and dendron, that form the microbubble shell.

**Scheme 1:**
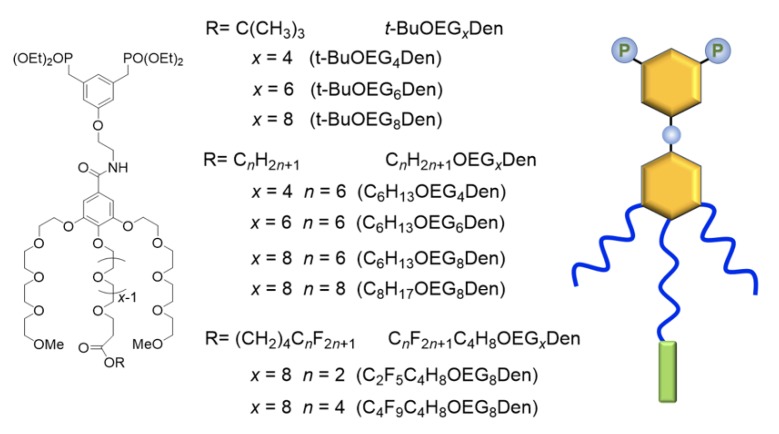
Molecular structure and schematic representation of the oligo(ethylene glycol) (OEG) dendrons investigated.

To this end, 10 dendrons were synthesized and investigated that featured two phosphonic esters and three OEG chains, including a longer one, connected by a phenoxyethylcarbamoylphenoxy group. The long OEG chain was fitted with a *t*-Bu, or a hydrocarbon chain (C_6_H_13_ or C_8_H_17_), or a partially fluorinated chain (C_2_F_5_C_4_H_8_ or C_4_F_9_C_4_H_8_) as the end group (Scheme 1). The number of ethylene glycol groups *x* was four, six, or eight. First, we have presented the behavior of these dendrons when spread on the surface of water, as studied by compression isotherms, compression/expansion cycles, and Brewster angle microscopy. Second, we have described the monolayer behavior of these OEG dendrons when mixed with a phospholipid, dimyristoylphosphatidylcholine (DMPC).

## 2. Results

### 2.1. Synthesis of OEG Dendrons

**Scheme 2:**
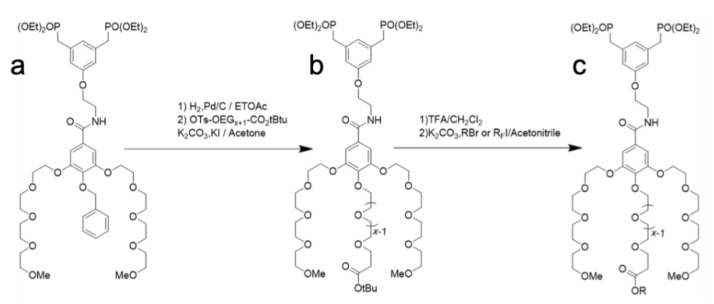
Multi-step synthesis of OEG dendrons, starting from (**a**) the BenzDen precursor, (**b**) introduction of *t*-Bu, and (**c**) alkylation with an alkyl or a perfluoroalkyl halide.

The dendrons were obtained via a multistep synthesis (Scheme 2). Starting from the BenzDen dendron precursor [24], the methodology involved the removal of the benzyl group and the introduction of the OEG chain in a basic medium, which provided the *t*-BuOEG*_x_*Den dendrons. After deprotection of the *t*-Bu group, the dendrons were alkylated using a hydrocarbon bromide or perfluoroalkyl iodide to give access to the dendrons fitted with a hydrocarbon chain (C*_n_*H_2*n*+1_OEG_8_Den) or a partially fluorinated chain (C*_n_*F_2*n*+1_C_4_H_8_OEG_8_Den).

### 2.2. Langmuir Monolayers of OEG Dendrons Carrying a t-Bu Group, an Alkyl, or a Partially Fluorinated Chain

#### 2.2.1. Isotherm Characteristics

**Figure 1:**
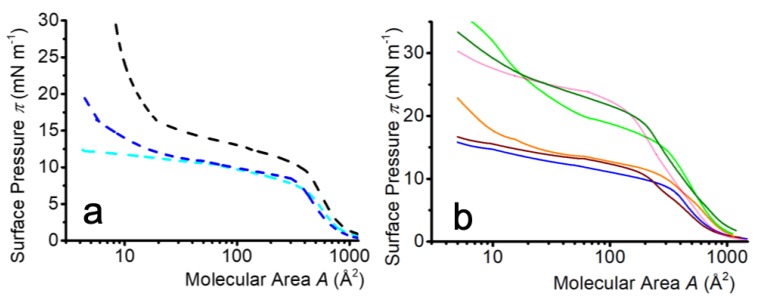
Compression isotherms at 25 °C of Langmuir monolayers of (**a**) *t*-BuOEG*_x_*Den (*x* = 4: light blue; *x* = 6: dark blue; *x* = 8: black) and (**b**) OEG dendrons fitted with hydrophobic chains (C_2_F_5_C_4_H_8_OEG_8_Den: light green; C_6_H_13_OEG_8_Den: orange; C_4_F_9_C_4_H_8_OEG_8_Den: dark green; C_8_H_17_OEG_8_Den: purple; C_6_H_13_OEG_6_Den: brown; and C_6_H_13_OEG_4_Den: blue).

The surface pressure/molecular area (*π*/*A*) isotherms at 25 °C of the 10 OEG dendrons investigated are presented in Figure 1. The isotherms presented the various regimes that can be ascribed to the OEG conformational transitions [7,19,20], by analogy to the behavior observed for diblock copolymers (for example, poly(styrene)-poly(ethylene oxide) [25]. At large molecular areas, liquid-expanded monolayers were observed in which the dendrons were anchored at the interface by the phenyl groups, while the OEG chains lay flat at this interface, forming a so-called “pancake” structure [19,26,27,28].

Within a homologous series (*t*-BuOEG*_x_*Den or C_6_H_13_OEG*_x_*Den), the extrapolated molecular area *A*_0_ increased with *x* (Table 1), which confirmed the pancake structure. A break (pseudo-plateau) was observed at a surface pressure *π*_p_. This break was associated with the progressive dissolution of the OEG chains in the water sub-phase [25,26,29]. The extension of the pseudo-plateau did not depend on *x*, likely because the increments in *x* were small. For *t*-BuOEGxDen, a significant increase of *π*_p_ with *x* was visible, however (*π*_p_: 6.0–6.5 mN m^−1^ for *x* = 4 or 6 and 9.6 mN m^−1^ for *x* = 8), in agreement with earlier reports [30,31,32]. Although collapse pressures could not be determined, the maximal pressures reached increased with *x* for *t*-BuOEG*x*Den and C*_n_*H_2*n*+1_OEG_8_Den (*n* = 6 and 8), reflecting better anchoring at the water surface.

For a given *x* value, *A*_0_ was found to be smaller and *π*_p_ higher for the dendrons fitted with the most hydrophobic end-groups, such as C_8_H_17_, C_4_F_9_C_4_H_8_, or C_2_F_5_C_4_H_8_ (Table 1). This suggested that the latter might counteract the anchoring of the OEG chains at the air/water interface by modifying their hydrogen bonds with water, and oppose their dissolution in the water phase. It is also likely that the hydrophobic end-groups promote the coiling of the OEG chain in the *para* position, thus hindering their dissolution in the aqueous sub-phase. It is noteworthy that the increase of *π*_p_ was more pronounced for C_2_F_5_C_4_H_8_OEG_8_Den than for C_6_H_13_OEG_8_Den, reflecting the higher hydrophobicity of the fluorinated moieties [33,34]. At low molecular areas (high coverage), only some of the isotherms showed a modest increase in *π* upon compression, reflecting the fact that a “brush-like” liquid condensed state, in which the OEG chains would be straightened in water, was not reached. 

#### 2.2.2. Isotherm Reversibility

**Figure 2:**
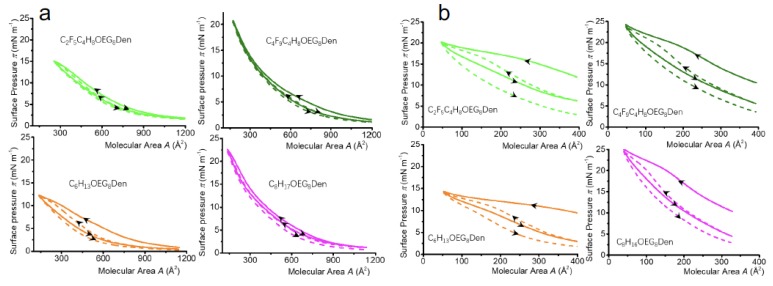
Compression–expansion cycles of Langmuir monolayers of OEG dendrons fitted with hydrophobic end-groups, recorded in (**a**) the liquid-expanded regime and (**b**) the plateau. The first and second compression–decompression cycles are indicated by solid and dashed lines, respectively.

In order to investigate whether the dendrons would expand again after compression, two compression–expansion cycles were performed for dendrons fitted with a hydrocarbon chain (C_6_H_13_ or C_8_H_17_) or a partially fluorinated chain (C_2_F_5_C_4_H_8_ or C_4_F_9_C_4_H_8_). Hysteresis cycles were recorded both in the liquid-expanded state of the monolayers (Figure 2a) and on the OEG chain conformational transition plateau (Figure 2b). For all dendrons but C_6_H_13_OEG_8_Den, the isotherms exhibited only minimal hysteresis, reflecting the stability of the monolayers when cycled in the fluid state. The difference in stability between the fluorinated dendron C_2_F_5_C_4_H_8_OEG_8_Den and its hydrocarbon analog C_6_H_13_OEG_8_Den confirmed the stabilizing effect of the C_2_F_5_ group that contributed to anchoring the monolayer at the interface, owing to the low interfacial tension of the fluorinated chains [35,36]. When the monolayers were successively compressed and expanded on the plateau, the isotherms presented significant hysteresis, which was likely due to the OEG chain conformational transition that induced a desorption of the dendrons from the interface.

#### 2.2.3. Brewster Angle Microscopy and Atomic Force Microscopy

**Figure 3:**
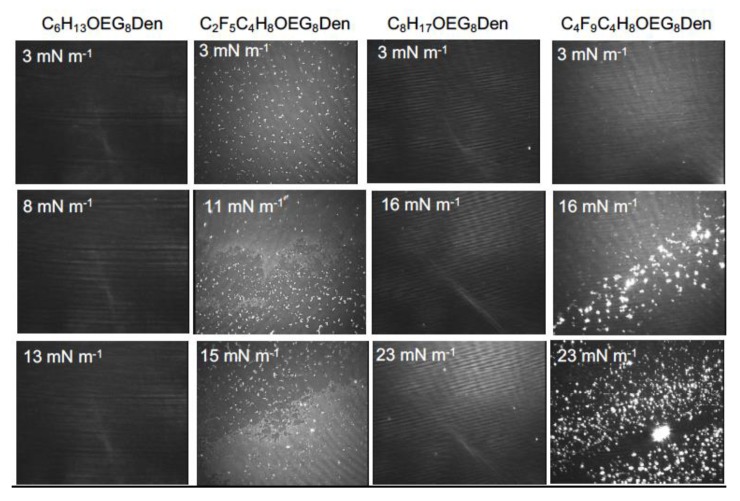
Brewster angle microscopy (BAM) images of the monolayers of OEG dendrons fitted with hydrogenated or fluorinated end-groups. Size of the images is 600 × 500 µm^2^.

The monolayers of OEG dendrons fitted with a hydrocarbon chain (C_6_H_13_ or C_8_H_17_) or a partially fluorinated chain (C_2_F_5_C_4_H_8_ or C_4_F_9_C_4_H_8_) were investigated by BAM and AFM (Figure 3). BAM images indicated that the hydrocarbon dendrons C_6_H_13_OEG_8_Den and C_8_H_17_OEG_8_Den formed fluid monolayers at all surface pressures. By contrast, the two fluorinated dendrons (C_2_F_5_C_4_H_8_OEG_8_Den and C_4_F_9_C_4_H_8_OEG_8_Den) formed crystalline-like domains from the beginning of the compression. These domains persisted throughout compression. A variation of the domain size was also observed, probably reflecting some coalescence.

### 2.3. Mixed Langmuir Monolayers of Phospholipid and OEG Dendrons

#### 2.3.1. Characteristics of the Compression Isotherms

**Figure 4:**
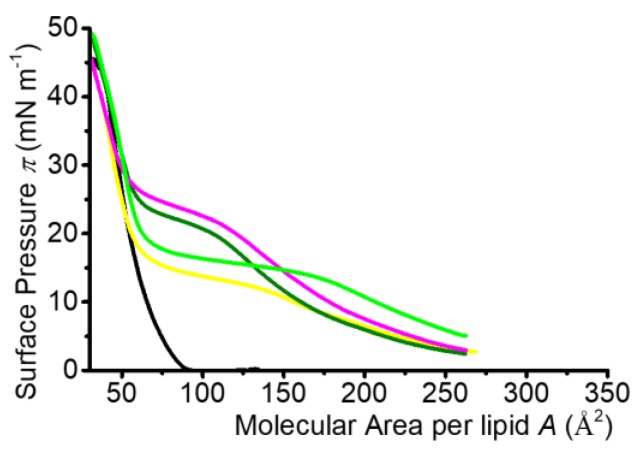
Compression isotherms of dimyristoylphosphatidylcholine (DMPC) (black) and DMPC/OEG dendron mixed Langmuir monolayers (DMPC/dendron molar ratio: 75:25; C_2_F_5_C_4_H_8_OEG_8_Den: green; C_6_H_13_OEG_8_Den: orange; C_4_F_9_C_4_H_8_OEG8Den: dark green; C_8_H_16_OEG_8_Den: purple). Temperature was 25 °C.

Compression isotherms and BAM images were recorded for mixed monolayers of DMPC and OEG dendrons carrying hydrocarbon chains (C_6_H_13_OEG_8_Den and C_8_H_17_OEG_8_Den), or partially fluorinated chains (C_2_F_5_C_4_H_8_OEG_8_Den and C_4_F_9_C_4_H_8_OEG_8_Den). A DMPC/OEG dendron molar ratio of 75:25 was set, this composition having been selected as optimal for the formulation of microbubbles [23]. Most commercially available microbubble-based contrast agents indeed possess a shell made of phospholipids [37,38,39]. The compression isotherms of DMPC and of the mixed monolayers are displayed in Figure 4. The DMPC monolayer presented a monotonous *π*/*A* curve, typical of a liquid-expanded state, throughout compression. The isotherms of the mixed monolayers presented a significant shift to larger molecular area and a pseudo-plateau, indicating that both components were present at the interface. The *π*_p_ values on the plateaus corresponded well to the *π*_p_ values observed for dendrons as the sole monolayer component. At small molecular areas, the isotherms of the mixtures coincided with those of the DMPC monolayer, indicating that dendrons were likely expelled from the phospholipid monolayer.

**Figure 5:**
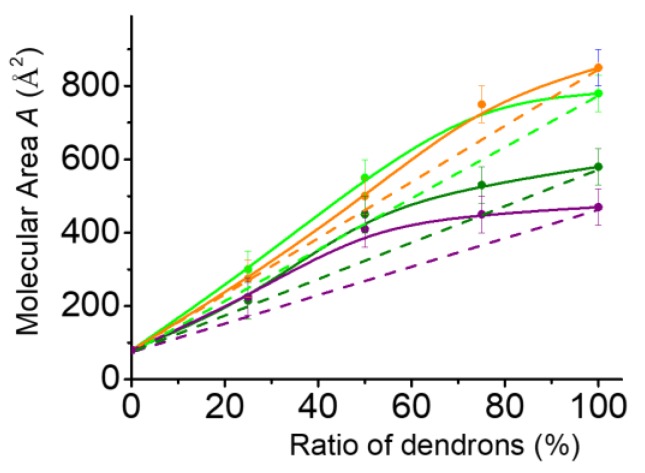
Variation of the extrapolated molecular area *A*_0_ as a function of molar ratio of dendrons in DMPC monolayers. C_6_H_13_OEG_8_Den (orange); C_2_F_5_C_4_H_8_OEG_8_Den (light green); C_8_H_17_OEG_8_Den (purple); and C_4_F_9_C_4_H_8_OEG_8_Den (dark green).

Next, the miscibility of DMPC and the dendrons in mixed monolayers was examined by plotting the variation of the molecular area *A*_0_ versus the dendron molar ratio (Figure 5). Positive deviations from ideality were determined using the additivity rule [40,41]. For dendrons fitted with the longest chains, the deviations were observed to affect monolayers with a larger dendron molar ratio. Partial fluorination of the hydrophobic chain also increased the deviation to ideality. This indicated limited miscibility with repulsive interactions between the monolayer components.

#### 2.3.2. Isotherm Reversibility

**Figure 6:**
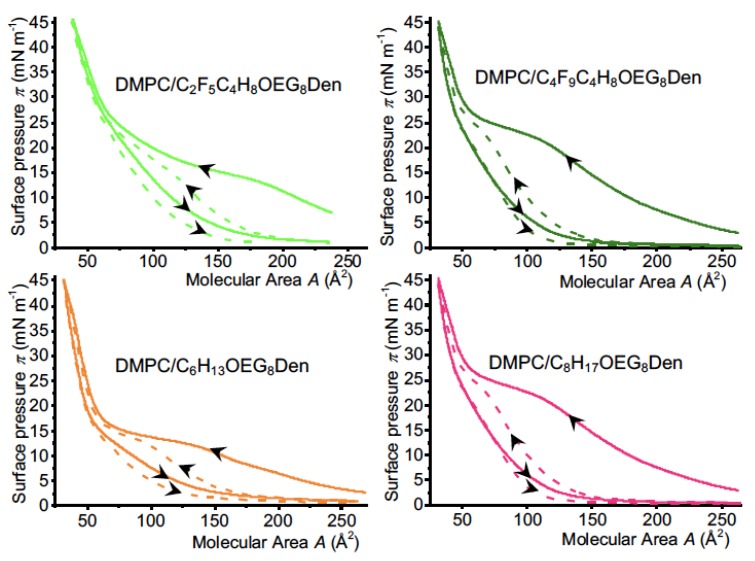
Hysteresis compression–expansion cycles of mixed DMPC/OEG dendrons Langmuir monolayers at the air/water interface (DMPC/dendron molar ratio: 75:25). First and second compression–expansion cycles are indicated by solid and dashed lines, respectively.

Mixed DMPC/OEG dendron monolayers were subjected to compression–expansion cycles in the transition regime (Figure 6). Strong hysteresis was observed during decompression for both fluorinated and hydrocarbon dendrons, which indicated that the monolayer components did not re-spread easily at the interface due to intermolecular interactions, suggesting the possible formation of crystalline-like domains. During the second compression *π* strongly decreased, which indicated that the dendrons were progressively expelled from the monolayers when the mixed monolayers were compressed at *π* > *π*_p._

#### 2.3.3. Brewster Angle Microscopy and Atomic Force Microscopy

**Figure 7:**
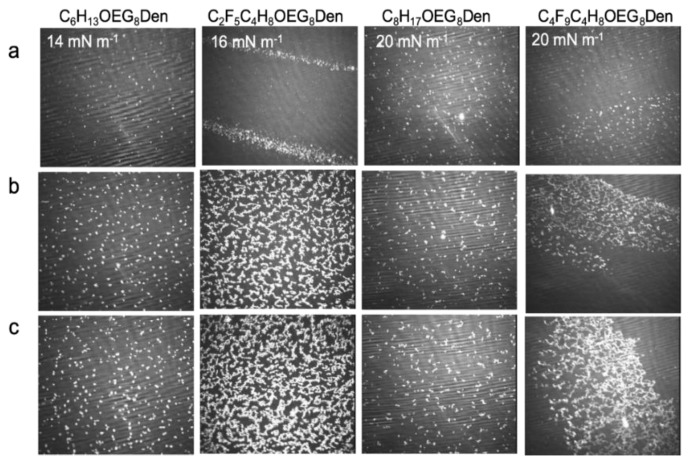
BAM images of DMPC/OEG dendron mixed Langmuir monolayers at the air/water interface (**a**) at the transition, (**b**) at 30 mN m^−1^, and (**c**) after collapse at 40 mN m^−1^.

Representative BAM images of the mixed DMPC/OEG dendron monolayers are displayed in Figure 7. The DMPC monolayer was in a liquid-expanded state throughout compression, which resulted in a featureless BAM image.

By contrast, the images of the mixed monolayers displayed domains (white features) for all dendrons and all surface pressures. These domains became more numerous as *π* increased. It is noteworthy that even the dendrons fitted with the hydrocarbon chains, which formed fluid monolayers when spread alone on the surface of water, formed domains when mixed with DMPC. The domains were particularly numerous in the case of C_2_F_5_C_4_H_8_OEG_8_Den, owing to the increased lipophobicity of the fluorinated chains.

**Figure 8:**
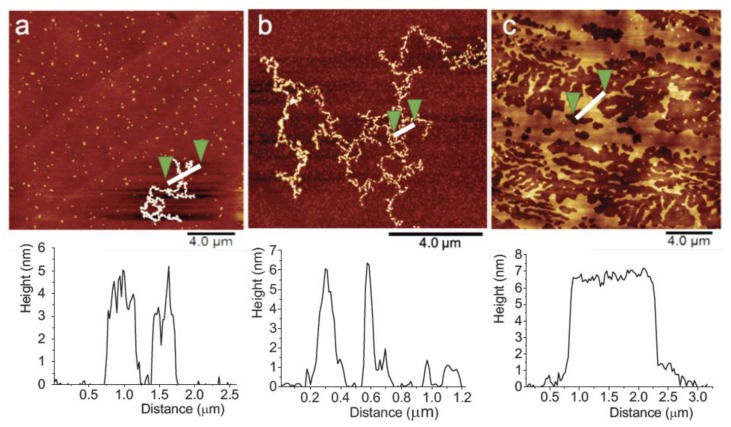
Atomic force microscopy (AFM) images and height profiles of mixed DMPC/C_2_F_5_C_4_H_8_OEG_8_Den monolayers (molar ratio: 75:25) after transfer onto silicon wafers at (**a**) 14 mN m^−1^, (**b**) 30 mN m^−1^, and (**c**) 44 mN m^−1^.

Atomic force microscopy was performed in order to get more information on the domains formed in the DMPC/C_2_F_5_C_4_H_8_OEG_8_Den (75:25 molar ratio) mixed monolayers. The micrographs showed that, when the monolayers were transferred on the plateau (14 mN m^−1^), small circular domains were predominantly formed, along with some rare coiled aggregates (Figure 8a). When *π* increased, the number of coiled aggregates and their size increased (Figure 8b). At 44 mN m^−1^, that is, near collapse, a dendritic pattern of dendrons was formed that quasi-totally covered the DMPC monolayer (Figure 8c). This indicated that the dendrons were progressively expelled from the phospholipid monolayer. The mean height of the aggregates above the DMPC monolayer was consistent with a bilayer of dendrons (~6 nm). Furthermore, it was observed that the height of the aggregates increased slightly, but significantly, with the surface pressure at which the monolayers were transferred. For example, the measured heights were 5.1, 6.3, and 7.0 ± 0.3 nm for transfer pressures of 14, 30, and 44 mN m^−1^, respectively (Figure 8c). This trend also supported the view that the dendron aggregates, which were initially embedded in the DMPC monolayer, were progressively expelled, self-assembled into strands, and eventually formed plaques that covered the monolayer.

## 3. Conclusions and Perspectives

Our main objective was to investigate the interfacial behavior of phospholipid-embedded OEG dendrons containing a *t*-Bu, a hydrocarbon, or a fluorinated end-group. These compounds are structurally close to the dendrons used to coat iron oxide nanoparticles developed for imaging and hyperthermia procedures. These dendronized magnetic nanoparticles are presently being investigated in combination with phospholipids to stabilize medical microbubbles. All the experiments conducted in this study indicated that OEG dendrons were expelled during compression, both from the air/water interface when they were spread as the sole component, and from DMPC monolayers, when they were co-spread at the interface with this phospholipid. When the surface density of dendrons triggered the OEG conformational transition, the dendrons were desorbed from the interface and expelled in the aqueous phase. This means that the three OEG chains of unequal lengths were not hydrophilic enough to enable the formation of a brush-like liquid condensed phase, as has been observed with PEG- and some OEGylated dendrons. These mixed phospholipid/dendron monolayers can be viewed as model interfaces of the monolayers that form the shells of medical microbubbles. In this regard, this work suggests that the propensity of OEG dendrons to be squeezed out from phospholipid monolayers could be exploited to facilitate the delivery of dendronized magnetic nanoparticles in vivo, and most particularly for the combined use of ultrasound and magnetic resonance tumor imaging modalities.

## 4. Materials and Methods 

### 4.1. Materials

1,2-dimyristoylphosphatidylcholine (DMPC) was purchased as a dry powder (99% purity) from Avanti Polar Lipids (Alabaster, AL, USA) and used as received. Water was purified using a Millipore system (surface tension 72.1 mN m^−1^ at 20 °C, resistivity: 18.2 MΩ cm).

### 4.2. Synthesis of the Dendrons

***t*-BuOEG*x*Den** (*x* = 4, 6, 8): Pd/C 10% (0.1 equiv.) was added to a solution of BenzDen (1.0 equiv.) in EtOAc (0.1 M). The heterogeneous solution was backfilled five times with an atmosphere of hydrogen, then vigorously stirred at room temperature overnight. The catalyst was filtered over Celite and the crude product was concentrated under reduced pressure and dissolved in acetone (0.1 M), after which K_2_CO_3_ (1.5 equiv.) and OTsOEG*_x_*CO_2_*t*-Bu (1.1 equiv.) were added. The resulting heterogenous solution was stirred at reflux for 16 h and cooled to room temperature. After filtration, the crude product was concentrated under reduced pressure. Chromatography on silica gel afforded the final product as a yellow oil.

**t-Bu-OEG_4_Den**: ^1^H NMR (500 MHz, CDCl_3_): δ (ppm): 7.11 (s, 2H), 6.88 (brs, 1H), 6.82 (s, 1H), 6.78 (d, *J* = 2.2 Hz, 1H), 4.22 (t, *J* = 4.7 Hz, 4H), 4.14 (t, *J* = 4.9 Hz, 2H), 4.02 (qt, *J* = 7.4 Hz, 8H), 3.85 (t, *J* = 5.1 Hz, 4H), 3.82–3.77 (m, 4H), 3.72–3.60 (m, 56H), 3.54–3.52 (m, 4H), 3.36 (s, 6H), 3.09 (d, 2*J*_P-H_ = 22.3 Hz, 4H), 2.50 (t, *J* = 6.2 Hz, 2H), 1.44 (s, 9H), 1.25 (t, *J* = 7.2 Hz, 12H). ^13^C NMR (125 MHz, CDCl_3_): δ (ppm): 170.9, 167.2, 152.4, 133.3, 129.5, 114.6, 107.3, 80.5, 72.3, 71.9, 70.6-70.5 (several peaks), 70.3, 69.7, 69.1, 66.9, 66.7, 62.1 (d, ^2^*J*_C-P_ = 6.6 Hz), 59.0, 39.5, 36.2, 33.6 (d, 1*J*_C-P_ = 138.4 Hz), 28.1, 16.4 (d, ^3^*J*_C-P_ = 5.4 Hz). ^31^P NMR (202 MHz, CDCl_3_): δ (ppm): 26.0.

***t*-Bu-OEG_6_Den**: ^1^H NMR (500 MHz, CDCl_3_): δ (ppm): 7.11 (s, 2H), 6.88 (brs, 1H), 6.82 (s, 1H), 6.78 (d, *J* = 2.2 Hz, 1H), 4.22 (t, *J* = 4.7 Hz, 4H), 4.14 (t, *J* = 4.9 Hz, 2H), 4.02 (qt, *J* = 7.4 Hz, 8H), 3.85 (t, *J* = 5.1 Hz, 4H), 3.82–3.77 (m, 4H), 3.72–3.60 (m, 56H), 3.54–3.52 (m, 4H), 3.36 (s, 6H), 3.09 (d, ^2^*J*_P-H_ = 22.3 Hz, 4H), 2.50 (t, *J* = 6.2 Hz, 2H), 1.44 (s, 9H), 1.25 (t, *J* = 7.2 Hz, 12H). ^13^C NMR (125 MHz, CDCl_3_): δ (ppm): 170.9, 167.2, 158.8, 152.5, 141.7, 133.3, 129.5, 124.4, 114.6, 107.3, 80.5, 72.3, 71.9, 70.7–70.3 (several peaks), 69.7, 69.1, 66.9, 66.7, 62.1 (d, ^2^*J*_C-P_ = 7.2 Hz), 59.0, 39.5, 36.2, 33.6 (d, 1*J*_C-P_ = 137.8 Hz), 28.1, 16.4 (d, ^3^*J*_C-P_ = 5.6 Hz). ^31^P NMR (202 MHz, CDCl_3_): δ (ppm): 26.0.

***t*-Bu-OEG6Den**: See characteristics in [24].

**C*_n_*H_2*n*+1_OEG_8_Den** (*n* = 6 and 8) and **C*_n_*F_2*n*+1_C_4_H_8_OEG_8_Den** (*n* = 2 and 4): TFA (5.0 equiv.) was added to a solution of *t*-BuOEG_8_Den (1.0 equiv.) in CH_2_Cl_2_ (0.1 M). The solution was stirred at room temperature for 4 h, concentrated under reduced pressure, dissolved in acetonitrile (0.1 M) before K_2_CO_3_ (2.0 equiv.) and the appropriate alkyl (or *F*-alkyl) halide (4.0 equiv., C_6_H_13_Br, C_8_H_17_Br, C_2_F_5_C_4_H_8_I, or C_4_F_9_C_4_H_8_I) was added. After stirring at reflux for 16 h and cooling to room temperature, the solid was filtered and the crude product was concentrated under reduced pressure. Chromatography on silica gel afforded the product as a yellow oil.

**C_6_H_13_OEG_8_Den**: ^1^H NMR (500 MHz, CDCl_3_): δ (ppm): 7.10 (s, 2H), 6.77 (s, 1H), 6.76 (s, 2H), 4.21 (t, *J* = 4.9 Hz, 4H), 4.18 (t, *J* = 5.1 Hz, 2H), 4.13 (t, *J* = 5.1 Hz, 2H), 4.07 (t, *J* = 6.8 Hz, 2H), 4.04–3.97 (m, 10H), 3.84 (t, *J* = 4.9 Hz, 4H), 3.81–3.76 (m, 6H), 3.73 (t, *J* = 6.4 Hz, 2H), 3.71–3.58 (m, 50H), 3.53–3.51 (m, 4H), 3.35 (s, 6H), 3.08 (d, ^2^*J*_P-H_ = 21.6 Hz, 4H), 2.58 (t, *J* = 6.5 Hz, 2H), 1.61 (qt, *J* = 9.5 Hz, 2H), 1.38—1.26 (m, 8H), 1.24 (t, *J* = 7.1 Hz, 12H), 0.88 (t, *J* = 7.1 Hz, 3H). ^13^C NMR (125 MHz, CDCl_3_): δ (ppm): 171.6, 167.3, 158.6, 152.4, 141.4, 133.3, 129.6, 124.0, 114.7, 107.3, 72.3, 71.9, 70.7–70.4 (several peaks), 69.7, 69.0, 66.7, 64.7 62.3, 59.0, 39.6, 35.1, 34.1, 33.0, 31.4, 28.5, 25.5, 22.5, 16.4, 14.0. ^31^P NMR (202 MHz, CDCl_3_): δ (ppm): 26.0.

**C_8_H_17_OEG_8_Den**: ^1^H NMR (500 MHz, CDCl_3_): δ (ppm): 7.10 (s, 2H), 6.88 (brs, 1H), 6.80 (s, 1H), 6.76 (s, 2H), 4.21 (t, *J* = 5.2 Hz, 4H), 4.18 (t, *J* = 5.2 Hz, 2H), 4.13 (t, *J* = 5.2 Hz, 2H), 4.06 (t, *J* = 6.8 Hz, 2H), 4.04–3.97 (m, 8H), 3.84 (t, *J* = 4.9 Hz, 4H), 3.80–3.77 (m, 4H), 3.74 (t, *J* = 6.7 Hz, 2H), 3.70–3.59 (m, 50H), 3.53–3.51 (m, 4H), 3.35 (s, 6H), 3.07 (d, ^2^*J*_P-H_ = 21.6 Hz, 4H), 2.58 (t, *J* = 6.7 Hz, 2H), 1.60 (qt, *J* = 9.5 Hz, 2H), 1.36–1.23 (m, 34H), 0.87 (t, *J* = 6.9 Hz, 3H). ^13^C NMR (125 MHz, CDCl_3_): δ (ppm): 171.6, 167.3, 158.8, 152.5, 141.7, 133.2 129.5, 124.1, 114.7, 107.4, 72.4, 71.9, 70.7–70.4 (several peaks), 69.7, 69.1, 66.7, 64.7 62.2, 59.0, 39.6, 35.1, 34.2, 33.1, 31.9, 29.6, 29.5, 29.3, 29.2, 26.3, 25.9, 22.7, 16.4, 14.1. ^31^P NMR (202 MHz, CDCl_3_): δ (ppm): 26.0 ppm.

**C_2_F_5_C_4_H_8_OEG_8_Den**: ^1^H NMR (500 MHz, CDCl_3_): δ (ppm): 7.10 (s, 2H), 7.00 (brs, 1H), 6.80 (s, 1H), 6.76 (s, 2H), 4.20 (t, *J* = 4.4 Hz, 4H), 4.17 (t, *J* = 4.6 Hz, 2H), 4.13–4.09 (m, 4H), 4.00 (qt, *J* = 7.4 Hz, 8H), 3.83 (t, *J* = 4.7 Hz, 4H), 3.79–3.75 (m, 4H), 3.73 (t, *J* = 6.4 Hz, 2H), 3.70–3.60 (m, 50H), 3.52–3.50 (m, 4H), 3.34 (s, 6H), 3.07 (d, ^2^*J*_P-H_ = 22.2 Hz, 4H), 2.58 (t, *J* = 6.6 Hz, 2H), 2.11–1.99 (m, 4H), 1.74–1.62 (m, 4H), 1.23 (t, *J* = 7.1 Hz, 12H). ^13^C NMR (125 MHz, CDCl_3_): δ (ppm): 171.5, 167.2, 158.6, 152.4, 141.4, 133.2, 129.5, 124.0, 114.7, 107.3, 72.3, 71.9, 70.6–70.4 (several peaks), 69.7, 69.0, 66.7, 66.5, 63.6, 62.2 (d, ^2^*J*_C-P_ = 6.9 Hz), 59.0, 39.5, 35.0, 34.1, 33.0, 30.9, 30.4, 30.2, 30.0, 29.7, 28.0, 17.1, 16.4 (d, ^3^*J*_C-P_ = 5.8 Hz). ^31^P NMR (202 MHz, CDCl_3_): δ (ppm): 26.3. ^19^F NMR (282 MHz, CDCl_3_): δ (ppm): −85.4, −118.3.

**C_4_F_9_C_4_H_8_OEG_8_Den**: ^1^H NMR (500 MHz, CDCl_3_): δ (ppm): 7.09 (s, 2H), 6.88 (brs, 1H), 6.79 (s, 1H), 6.75 (s, 2H), 4.20 (t, *J* = 4.9 Hz, 4H), 4.17 (t, *J* = 4.9 Hz, 2H), 4.11–4.09 (m, 4H), 4.03–3.96 (m, 8H), 3.82 (t, *J* = 5.1 Hz, 4H), 3.79–3.75 (m, 4H), 3.73 (t, *J* = 6.5 Hz, 2H), 3.69–3.56 (m, 48H), 3.51–3.50 (m, 4H), 3.33 (s, 6H), 3.07 (d, ^2^*J*_P-H_ = 21.6 Hz, 4H), 2.57 (t, *J* = 6.6 Hz, 2H), 2.18–2.02 (m, 4H), 1.74–1.63 (m, 4H), 1.23 (t, *J* = 7.0 Hz, 12H). ^13^C NMR (125 MHz, CDCl_3_): δ (ppm): 171.5, 167.2, 158.6, 152.4, 141.5, 133.3, 129.5, 124.0, 114.6, 107.3, 72.2, 71.9, 70.7–70.4 (several peaks), 69.7, 69.1, 66.7, 63.6 62.2, 59.0, 39.5, 35.0, 34.2, 33.1, 30.4, 28.0, 17.0, 16.4. ^31^P NMR (202 MHz, CDCl_3_): δ (ppm): 26.0 ppm. ^19^F NMR (480 MHz, CDCl_3_): δ (ppm): −126.0, 124.5, −114.5, −81.0.

### 4.3. Langmuir Monolayers

The surface pressure (*π*) versus molecular area (*A*) isotherms were recorded using a Langmuir minitrough (KSV NIMA, Finland) equipped with two movable barriers (initial area: 365 × 75 mm^2^, compression speed: 10 cm^2^ min^−1^, which corresponded to a reduction of the total area of ∼3.6% min^−1^). *π* was measured using the Wilhelmy plate (paper) method. The trough was maintained at 25 ± 0.5 °C. Solutions of OEG dendrons (1 mmol L^−1^) in chloroform were spread on the surface of water (320 mL). Subsequently, 15 min was allowed for chloroform to evaporate and the film to equilibrate before compression was initiated. All the experiments were performed at least three times. Since our Langmuir trough only allowed for a surface area compression of about 10, isotherms were recorded in three separate experiments.

### 4.4. Atomic Force Microscopy (AFM)

The monolayers of dendrons or DMPC/dendron mixtures were compressed up to the desired surface pressure and transferred at constant surface pressure onto silicon wafers using the Langmuir–Blodgett technique (one monolayer transferred; lift speed: 1 mm min^−1^). Silicon wafers were cleaned for 30 min in a sonication bath containing ethanol/milliQ water (1:1 vol:vol), followed by 2 min in a plasma cleaner. The LB films were immediately analyzed by AFM after preparation. Images of the transferred films were recorded in tapping mode (AFM multimode 8, Bruker, Santa Barbara, CA, USA). The cantilever (Budget Sensors) had a 3–10 nm radius silicon tip. The typical resonance frequency was 300 kHz and the spring constant was 40 N m^−1^. At least three different samples were analyzed and several areas were scanned on the silicon wafer for each sample. Errors of the measurements along the z axis were estimated to be ± 0.5 nm.

### 4.5. Brewster Angle Microscopy (BAM)

When a laser beam polarized parallel to the plane of incidence hits the air/water interface at an angle of 53.15°, which is called the Brewster angle (= arctan *n*_water_/*n*_air_, with *n* the refractive index), there is essentially no light reflected. However, the presence of a monolayer at the interface alters the Brewster conditions, which results in some light being reflected. The intensity of the reflected light is a function of film thickness and refractive index. With an optical microscope set at the Brewster angle, the water surface appears dark and the thin film brighter. In this way, BAM allows the direct observation of some morphological characteristics of monolayers. A Bam2Plus microscope (NFT, Gottingen, Germany) equipped with a KSV Minitrough Langmuir system (320 × 75 mm^2^) was used for the experiments. The volumes of dendron solution (1 mM) and DMPC/dendron mixtures (molar ratio: 75:25) deposited on the surface were 5 µL and 20 µL, respectively. Snapshots were captured when the monolayers were compressed up to desired pressures at a compression speed of 1.5 cm^2^ min^−1^. The scale of the images is 600 × 500 µm.

## Figures and Tables

**Table 1:** Characteristics of monolayers of OEG dendrons. Extrapolated molecular area in the liquid-expanded phase (*A*_0_) and surface pressure on the plateau (*π*_p_).

Dendrons	*A*_0_ (Å^2^) ± 50 Å^2^	*π*_plateau_ (mN m^−1^) ± 1 mN m^−1^
*t*-BuOEG_4_Den	750	6.0
*t*-BuOEG_6_Den	850	6.5
*t*-BuOEG_8_Den	900	9.6
C_6_H_13_OEG_4_Den	700	7.2
C_6_H_13_OEG_6_Den	720	9.9
C_6_H_13_OEG_8_Den	850	9.1
C_2_F_5_C_4_H_8_OEG_8_Den	780	13.1
C_8_H_17_OEG_8_Den	470	18.3
C_4_F_9_C_4_H_8_OEG_8_Den	580	17.7
